# Use of folding modulators to improve heterologous protein production in *Escherichia coli*

**DOI:** 10.1186/1475-2859-8-9

**Published:** 2009-01-27

**Authors:** Olga Kolaj, Stefania Spada, Sylvain Robin, J Gerard Wall

**Affiliations:** 1 Department of Chemical and Environmental Sciences and Materials and Surface Science Institute, University of Limerick, National Technology Park, Limerick, Ireland; 2 Department of Microbiology, School of Natural Sciences, National University of Ireland, Galway, Galway, Ireland; 3 National Centre for Biomedical Engineering Science, National University of Ireland, Galway, Galway, Ireland

## Abstract

Despite the fundamental importance of *E. coli *in the manufacture of a wide range of biotechnological and biomedical products, extensive process and/or target optimisation is routinely required in order to achieve functional yields in excess of low mg/l levels. Molecular chaperones and folding catalysts appear to present a panacea for problems of heterologous protein folding in the organism, due largely to their broad substrate range compared with, *e.g*., protein-specific mutagenesis approaches. Painstaking investigation of chaperone overproduction has, however, met with mixed – and largely unpredictable – results to date. The past 5 years have nevertheless seen an explosion in interest in exploiting the native folding modulators of *E. coli*, and particularly cocktails thereof, driven largely by the availability of plasmid systems that facilitate simultaneous, non-rational screening of multiple chaperones during recombinant protein expression. As interest in using *E. coli *to produce recombinant membrane proteins and even glycoproteins grows, approaches to reduce aggregation, delay host cell lysis and optimise expression of difficult-to-express recombinant proteins will become even more critical over the coming years. In this review, we critically evaluate the performance of molecular chaperones and folding catalysts native to *E. coli *in improving functional production of heterologous proteins in the bacterium and we discuss how they might best be exploited to provide increased amounts of correctly-folded, active protein for biochemical and biophysical studies.

## Background

*E. coli *is preferred for heterologous protein production because of its fast growth, simple fermentation, uncomplicated nutritional and sterility requirements, and extensive characterisation [1]. Despite its widespread use [2], however, many heterologous proteins are produced as insoluble aggregates in cytoplasmic or periplasmic inclusion bodies, while the membrane leakiness and cell lysis associated with making heterologous proteins in *E. coli*, leading to significantly reduced yields, have also been well documented [3,4].

The discovery of molecular chaperones and folding catalysts appeared to present a panacea for protein aggregation and cell lysis problems in *E. coli *[5-7]. Molecular chaperones prevent aggregation by binding exposed hydrophobic moieties in unfolded, partially folded or misfolded polypeptides and traffic molecules to their subcellular destination, while folding catalysts catalyse potentially rate-limiting steps in the folding process, such as peptidyl bond isomerisation or disulfide bond formation. Painstaking investigation of these molecules has met with disparate results to date as simple overexpression of a single modulator in the absence of its accessory molecules frequently sees no positive effect – and may simply increase the production load to the further detriment of the recombinant target. In the absence of an ability to predict the relevant bottleneck in *E. coli*, therefore, an increasingly common approach by researchers is the use of chaperone "cocktails", which is facilitated by the recent availability of a number of plasmid systems that can be used to co-produce up to 6–7 folding modulators with a heterologous protein [8,9].

A universal system of producing active, recombinant proteins in *E. coli *remains a core objective of the biotechnology industry. In this paper, we review progress in exploiting folding modulators from *E. coli *to improve the functional production of heterologous proteins.

## Folding in the cytoplasm

**Figure 1:**
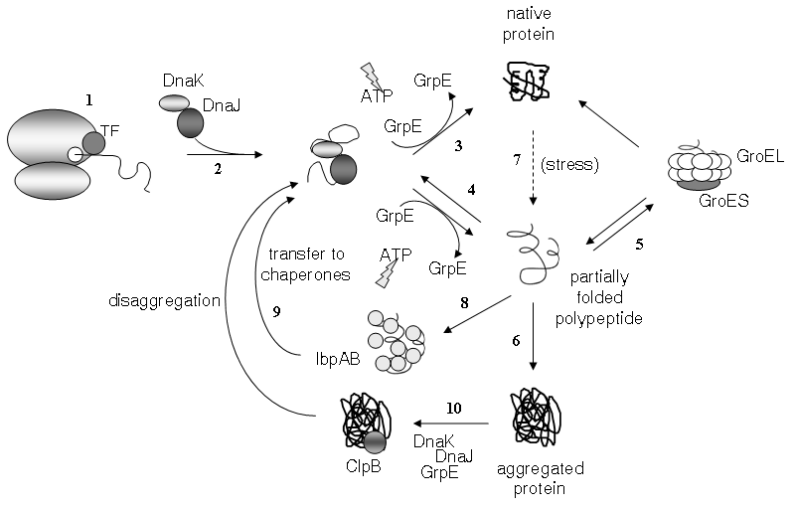
**The process of protein folding in the *E. coli *cytoplasm**. Nascent polypeptides encounter trigger factor (TF) chaperone upon emerging from the ribosomal exit tunnel (1). They can also be captured by DnaK (2) which cooperates with its cofactor DnaJ and nucleotide exchange factor GrpE to promote folding to a native (3) or partially folded conformation. The latter may be re-captured by DnaK (4) and possibly TF to repeat this folding cycle until it reaches its native state, interact with GroEL-GroES (5) to complete folding to its native conformation, or undergo aggregation (6). Upon heat stress, partial unfolding of thermolabile proteins can occur, resulting in exposure of aggregation-prone hydrophobic regions (7). sHsps such as IbpA and IbpB act to "hold" partially unfolded proteins for transfer to Hsp70 and Hsp60 chaperones DnaK and GroEL (8,9), while disaggregation of unfolded proteins is carried out by ClpB in cooperation with the Hsp70 family (10), followed again by their transfer to the DnaK/DnaJ/GrpE machinery for completion of folding.

The major groups of molecular chaperones involved in protein folding in the *E. coli *cytoplasm are the peptidyl-prolyl *cis*-*trans *isomerase and molecular chaperone trigger factor (TF) and members of the *h*eat *s*hock *p*rotein Hsp70 and Hsp60 families, in addition to ClpB that disaggregates polypeptide aggregates and the small heat shock proteins. The process of polypeptide folding in the *E. coli *cytoplasm and the chaperones involved are summarised in Figure 1.

### Cytoplasmic PPIases

Most peptide bonds are synthesised in the *trans *conformation at the ribosome but both the *cis *and *trans *conformations are accessible in peptides. As a result, *cis*-*trans *isomerisation of peptidyl-prolyl bonds is a potentially rate-limiting step in the folding process (reviewed in [10]). TF is a cytoplasmic enzyme with PPIase activity and the first chaperone to interact with nascent polypeptides at the ribosome (Figure 1). While it binds the ribosome at a 1:1 ratio [11,12], most TF in the cell is proposed to exist in a monomer-dimer equilibrium in the cytosol [13], in which the dimeric state is postulated to have a specific mechanistic role in posttranslational protein folding [14]. The activity of TF in supporting polypeptide folding overlaps at least in part with that of the downstream Hsp70 protein DnaK, as evident from the fact that the loss of either chaperone can be tolerated by *E. coli *but their combined deletion is lethal under normal growth conditions [15].

#### Overproduction of trigger factor

As TF displays both chaperone and PPIase activity *in vivo *and *in vitro *[16,17], it has been the subject of considerable interest in co-production experiments – despite the fact that the majority of newly synthesised polypeptides do not require it for *de novo *folding [18]. Nevertheless, TF co-production led to a 4-fold increase in expression of an anti-digoxin Fab antibody fragment in the *E. coli *cytoplasm [19] and a higher yield of soluble human ORP150 and lysozyme [17], with further improvements in expression and solubility achieved in the latter case by co-overproducing the Hsp60 GroESL. Similarly, a 3.8-fold increase in the solubility of human translation initiation factor eIF2α was noted upon TF and GroESL co-overproduction [20], though the addition of GroESL to successful TF co-production experiments can also be un- [17] or counter-productive [21].

TF co-production can also be synergistic with that of Hsp70 family members DnaK-DnaJ-GrpE, as observed in a temperature-dependent effect on guinea pig liver transglutaminase production [22] and vasostatin [23], which may be linked to TF's reported *in vivo *role in enhancing cell viability at low temperatures [24]. In an attempt to determine the mode of action of TF, mutants with very low PPIase activities were found to enhance soluble production of an adenylate kinase to the same extent as wildtype TF [25], indicating that the effect of TF on at least some recombinant proteins may be due to its chaperoning rather than isomerisation activity. The observation that human FKBP12, which has PPIase but no chaperone-like activity, did not improve expression of a thiosulfate sulfurtransferase enzyme that benefitted from co-production of an archaeal FKBP [26] provides additional evidence that many of the positive effects of PPIases in foreign protein production may relate to their chaperone-like rather than their isomerisation activity.

### Hsp70 family

The *h*eat *s*hock *p*rotein 70 (Hsp70) family of proteins are ubiquitous, highly conserved molecules whose predominant unifying feature is the ability to bind short, linear hydrophobic regions of polypeptides [27,28]. In addition to their role under heat stress, they assist in folding of newly translated polypeptides and subcellular trafficking of polypeptides under normal physiological conditions. Members of the family contain an ATPase domain and a more variable, peptide-binding domain and polypeptide binding and release is carried out in a cycle between an ATP-bound DnaK molecule with low substrate affinity and a high substrate affinity, ADP-bound state [29].

The activity of DnaK is dependent on the proteins DnaJ and GrpE in *E. coli*. DnaJ contains a highly conserved N-terminal region that interacts with DnaK, followed by a glycine/phenylalanine-rich region, a zinc finger domain that mediates polypeptide binding, and a variable C-terminal domain [29-31]. Following transient association between DnaK and DnaJ with concomitant ATP hydrolysis, GrpE catalyses the release of ADP from the DnaK-ADP-peptide complex. In this state, ATP is rapidly rebound by DnaK, leading to release of the bound polypeptide and of GrpE. This cycle of binding and release may be repeated many times, with the released peptide either recommencing the DnaJ/DnaK cycle, being transferred to the chaperonin GroEL, undergoing further folding steps to reach its native state, or aggregating (Figure 1; [32]).

#### Overproduction of DnaK-DnaJ-GrpE with cytoplasmic proteins

DnaK-DnaJ-GrpE chaperones are most commonly overproduced with cytoplasmic recombinant proteins, due to their own location in the cytoplasm. This approach has enabled the successful production of a number of proteins otherwise produced mainly or exclusively as inclusion bodies, such as a single-chain antibody fragment (scFv; [33]), human tyrosine kinases Csk, Fyn and Lck [34], an *Acinetobacter *cyclohexanone monooxygenase [35], and a cedar pollen allergen [36]. DnaK-DnaJ without GrpE have also been reported to increase production (red sea bream transglutaminase; [37]), suppress aggregation (human SPARC – secreted protein acidic and rich in cysteine; [38]), improve solubility (*Agrobacterium *D-hydantoinase; [39]), and increase the activity (β-galactosidase fusion protein; [40]) of numerous recombinant proteins. This improved production is generally due to increased solubility of recombinant targets rather than an increase in cellular production levels, though Nishihara and co-workers [17] reported a *decrease *in total murine endostatin concomitant with increased levels of soluble protein upon DnaK-DnaJ-GrpE overproduction. It should, however, be noted that increased solubility is not always accompanied by an increase in protein quality and so determination of solubility may not always provide an accurate picture of correct folding, as reported in a study of the effects of DnaK levels on a misfolding-prone GFP fusion protein [41,42].

Conversely, DnaK-DnaJ have little effect on the solubility and negative effects on the production and activity of numerous proline-rich targets [17,39,43], which emphasises the benefits of attempting to "match" chaperones to hypothetical bottlenecks in target protein production. Other workers have reported that protein aggregation could be prevented when DnaK-DnaJ-GrpE were co-expressed at 2–3 times wild type levels but that higher chaperone concentrations resulted in a reduced yield of recombinant protein [36]. These results highlight a recurring theme in this field, that chaperone overproduction must be regulated to meet the additional needs of the host cells, rather than serving to add to cellular stress through the high-level production of an irrelevant protein product [44].

The relatively recent availability, both commercial and non-commercial, of sets of *E. coli *chaperone co-production plasmids carrying the *groESL*, *dnaKJ grpE*, *tig *and other chaperone genes, frequently under independently regulated promoters, has led to numerous co-production analyses of Hsp70 proteins in combination with GroESL and trigger factor in particular. The successes reported with a variety of molecules from combining chaperones in this manner [17,44-47] and the ease of carrying out such broad screens means this type of approach will continue to provide an obvious starting point for researchers looking to improve expression of otherwise intransigent proteins.

#### Overproduction of Hsp70 family members with periplasmic proteins

Hsp70 co-production has also been employed to beneficial effect with heterologous proteins produced in the *E. coli *periplasm, apparently by increasing the solubility of the protein precursor prior to membrane translocation. A 100-fold increase in the yield of a scFv antibody fragment was observed upon co-producing DnaK-DnaJ-GrpE [48], while export of human granulocyte-colony stimulating factor [49], granulocyte-macrophage colony-stimulating factor and interleukin-13 [50] were greatly improved upon production of DnaK and DnaJ. In all cases, the amount of total cellular protein remained unchanged. A variation on this approach saw export of DnaJ itself to the *E. coli *periplasm, leading to dramatic (37- to 170-fold) increases in the functional periplasmic yields of a tissue plasminogen activator (tPA) variant and of proinsulin [51] – though no positive effect was found on a scFv-gene 3 protein fusion in the same study.

### Hsp60 family

The Hsp60 family is the most studied of all the chaperone families and is composed of GroEL and its homologues in prokaryotes, chloroplasts and mitochondria, and the TRiC/TCP-1 family in the eukaryotic cytosol. GroEL is characterised by a fascinating double ring-shaped structure composed of 14 identical subunits, stacked in 2 back to back heptameric rings, which together form a hollow cylinder containing a nucleotide binding site facing into the central channel [52]. GroEL acts by binding unfolded polypeptide at either of the outer ends of its inner cavity through hydrophobic interactions [53,54]. This is followed by capping of the cavity by its Hsp10 family co-chaperonin GroES, which exists as a single heptameric ring with a hollow dome-shape structure [55] to create a closed environment, with a capacity of approximately 86 kDa [56], in which substrate folding is favoured. Cycles of peptide binding and release are driven by ATP binding and hydrolysis, promoting a structural stretching of the guest protein until a sufficiently native state is reached such that exposed hydrophobic regions are no longer available to be bound in the GroEL cavity [57]. The demonstration that GroESL mediated folding of an 82-kDa aconitase protein that could not be encapsulated in the central GroEL cavity led more recently to the identification of a less efficient *trans *mechanism of polypeptide folding by GroEL, in which polypeptides are not encapsulated and the chaperone appears to act more as a holdase, suppressing off-pathway aggregation reactions, than as a foldase ([58]; reviewed in [59]).

#### Overproduction of GroESL with cytoplasmic, periplasmic proteins

Overproduction of GroESL has proven a highly productive approach to overcoming polypeptide folding problems in *E. coli*, allowing the soluble production of many recombinant proteins which are otherwise produced exclusively or almost exclusively in inclusion bodies. These include proteins as diverse as human thromboxane synthase [60], nicotinoprotein formaldehyde dismutase from *Pseudomonas putida *F61 [61], human oxygen-regulated protein ORP150 and human lysozyme [17], a human iron-regulatory protein [62], a putative bacterial dehydratase [63], β-glucosidases from *Cellovibrio gilvus *and *Agrobacterium tumefaciens *[64], murine c-Myb, cAMP response element-binding protein 1, p53 tumour suppresor gene product, *Xenopus mos *proto-oncogene product [65], bacterial magnesium transporter CorA [66] and triazine hydrolase from *Arthrobacter aurescens *TC1 [67]. A sample of proteins whose total or functional yield in the *E. coli *cytoplasm is merely *increased *upon GroESL overproduction, meanwhile, can be found in Table 1[19,21,36,39,43,58,61,64,68-101].

**Table 1:** Proteins whose total and/or functional yields increase upon co-production of GroESL

*Recombinant protein*	*Effect of co-production of GroESL chaperones*	*Reference*
Human procollagenase	GroESL increased production levels by 10-fold, solubility and half-life	[68]

p50^csk ^Protein-Tyrosine Kinase	Co-production enhanced solubility and activity of the protein by up to >50%	[69]

*Candida albicans *PMI metalloenzyme	2-fold increase in protein solubility	[70]

*α *and β subunits of human propionyl-CoA carboxylase (PCC)	Several hundred-fold increase in PCC specific activity; most of the protein produced in soluble form	[71]

Human electron transfer flavoprotein (ETF)	Co-production required for stable expression of ETF α G116R mutant	[72]

*β*-glucosidase from *Cellovibrio gilvus *and *Agrobacterium tumefaciens*	Co-production resulted in slower growth rate and reduced yield but increased solubility of the proteins by 20–60% at 37°C and up to 70% at 25°C	[64]

Cryj2 Japanese cedar pollen	Increased yield and solubility of expressed protein; 4-fold stabilisation of the protein in the presence of a 10-fold chaperone excess	[36]

Human kinase inhibitor-GST fusion	Solubility of otherwise mostly insoluble protein enhanced by 5–6-fold	[73]

Eukaryotic phenylalanine ammonia-lyase	Dramatically improved yield and activity of the protein after engineering of gene to remove *E. coli *rare codons	[74]

Bovine adrenodoxin reductase (AdR)	Increased soluble AdR yield to 10 mg/l, compared with 4 mg/l with Hsp70	[75]

Cyanobacterium transcription factor	3–4-fold increase in solubility	[76]

*Thermococcus litoralis *4-α-glucanotransferase (GTase)	Co-production of GroESL with tRNA_AGA _and tRNA_AGG _led to 5-fold increase in GTase activity in soluble fraction; yield otherwise lower and 60% insoluble	[77]

*Agrobacterium radiobacter *carbamoylase	4-fold increase in activity due to improved solubility	[39]

Human cytochrome P450 3A7 (CYP3A7)	Increased expression levels and activity of the otherwise inactive protein	[78]

Decarboxylase component of human α-keto acid dehydrogenase complex	Co-production of GroEL or GroES resulted in increase in decarboxylase activity by 500-fold and 30-fold, respectively	[79]

Maize plastidic protoporphyrinogen IX oxidase (PPO)	6-fold increase in soluble PPO yield	[80]

Manganese catalase from *Thermus *sp.	Increased solubility (up to 50%) with GroESL	[81]

p66 and p51 subunits of HIV-1 RTase	Yield and nucleic acid affinity increased by 4–5- and 1.6-fold, respectively	[82]

Anti-digoxin Fab antibody fragment	4-fold increase in solubility of the Fab produced in *E. coli *Origami strain	[19]

*Agrobacterium tumefaciens *D-carbamoylase (DCB)	Increase in solubility of DCB up to 60% and activity by 6.2-fold at 28°C; at 25°C protein solubility increased to 75% and activity by 4.5-fold	[83]

Guinea pig NADPH:quinone oxidoreductase	3-fold increase in solubility	[84]

Aconitase	Solubility and activity increased to 40% and by 1.5-fold, respectively	[58]

*Rhodococcus erythropolis *desulfinase	Solubility of the protein increased up to 40–50% and activity by 25-fold	[85]

*E. coli *glutamate racemase (GluR)	Growth of host cells improved; 2.2-fold increase in yield of active GluR	[86]

*Pseudomonas putida *F61 nicotinoprotein formaldehyde dismutase (NDF)	With *tac *promoter, increased solubility (up to 80%) and 6-fold higher enzyme activity; lesser effect when NDF expressed under the *lac *promoter	[61]

Human PP2A methyltransferase	24-fold increase in solubility	[87]

Oligo-1,6-glucosidase from *Bacillus thermoglucosidasius*	Specific activity increased by 44%, 56% and 56% with co-production of GroES, GroEL and GroESL, respectively	[43]

Cyclodextrin gluanotransferase (CGTase) from *Bacillus macerans*	Increase in solubility and activity of CGTase by 12% and 1.5-fold, respectively, at 37°C and by 22% and 1.3-fold, respectively, at 25°C	[88,89]

*Rhizobium *sp. α-1,6-fucosyltransferase	At 30°C, improved folding and an increase in specific activity by 1.76-fold	[90]

Mouse CYP27B1 protein	10-fold increase in the yield of stable and active protein	[91]

Pyridoxine 4-oxidase (PNO) from *Microbacterium luteolum*	No benefit of GroESL at 37°C; co-production at 23°C enhanced solubility and specific activity of PNO by 1.9-fold and 3.9-fold, respectively	[92]

Pyridoxal 4-dehydrogenase from *Microbacterium luteolum*	Co-production at 20°C led to reduced amounts of insoluble protein and increased specific activity by 9.1-fold	[93]

*Alcaligenes xylosoxydans *N-acyl-D-amino acid amidohydrolases	Enzymatic activity of the proteins increased from 7.8 to 72.4 U/mg and 7.1 to 22.7 U/mg, respectively, at 30°C	[21]

Human aromatase (P450arom, CYP19) NmA264C and NmA264R mutants	No improvement with NmA264C; production of NmA264R greatly enhanced (up to 400 nmol/l)	[94]

scFv specific for c-Met	Solubility increased 2-fold in *E. coli *Origami2(DE3) but not in BL21(DE3)	[95]

Yeast mitochondrial aconitase	Increased solubility at 25°C with no change in total yield	[96]

human prolyl hydroxylase isoenzyme	2-fold increase in solubility when produced at 30°C	[97]

Pig liver esterase γ-isoenzyme (PLE)	Enhanced yield of soluble and active PLE in *E. coli *Origami (DE3)	[98]

Soybean seed ferritin complex	Increased solubility of H-1 subunit from 4 to 39% and H-2 subunit from 19 to 85%	[99]

Human 11β hydroxylase	20- to 40-fold increase in yield in half the production time	[100]

Human glucose 6-phosphate dehydrogenase (G6PD) and mutants	Negligible effect on expression of wild type G6PD but activities of two mutants were enhanced by 48–160% and 39–118% at 37°C and 31°C, respectively	[101]

In spite of this impressive track record and the fact that GroEL has been demonstrated to support the folding of a majority of newly translated polypeptides in *E. coli *[54], GroESL overproduction is still not the much sought-after magic bullet for heterologous protein folding in *E. coli*. There are numerous reports of GroESL failing to improve protein solubility [102] or rescue recombinant proteins from inclusion bodies [103], even where co-production of Hsp70 family members was successful [22,37,48]. Overproduction of GroESL has also been found to lead to reduced enzyme activity [21] and lower viability of host cells during protein production [48]. These failings may reflect a degree of polypeptide specificity on the part of GroESL, as potentially evident in its differing effects on the expression of two human aromatase variants that differ only by a single amino acid residue [94]. Similarly, as discussed above with Hsp70 family members, GroESL overproduction has notably failed to improve the production of proteins with complex disulfide patterns [38,104,105] or in which peptidyl-prolyl *cis*-*trans *isomerisation is limiting [106] as the production bottleneck in such cases presumably lies outwith the remit of its chaperoning role.

Co-overproduction of GroESL with DnaK-DnaJ-GrpE and/or TF has led to numerous notable successes over those achievable with GroESL alone, such as with a human translation initiation factor [20], human oxygen-regulated protein ORP150 and human lysozyme [17], a D-aminoacylase [21] and, in temperature-dependent effects, with a GST-human vasostatin fusion protein [23] and human endostatin [45], all in combination with TF. Combining GroESL with DnaK-DnaJ-GrpE has proven significantly less fruitful, with numerous examples of losses (up to total) of positive effects on solubility or activity upon addition of the second chaperone family to the experimental setup [21,33,48,107]. As these multi-chaperone experiments usually have the singular objective of increasing target protein yields, however, they typically lack the detailed mechanistic studies necessary to delineate the effects of individual chaperones. In one attempt to delineate the respective roles of the Hsp60 and Hsp70 families in the cytoplasmic production of a penicillin acylase (PAC) precursor (proPAC), however, Xu and co-workers reported that the effect of GroESL co-production was to prevent intracellular proteolysis while DnaK-DnaJ-GrpE led to improved solubilisation of proPAC and improved PAC maturation [108].

While some success has resulted from co-producing chaperones such as DnaK with periplasm-destined recombinant proteins, comparably little success has accrued with GroES and GroEL. Thus it appears that, while GroESL overproduction represents a prime choice for investigation of folding defects of recombinant proteins expressed in the cytoplasm, it is typically unable to overcome bottlenecks associated with periplasmic production.

#### Overproduction of Hsp60 and Hsp70 members with membrane proteins

Recombinant production of membrane proteins in *E. coli *presents very particular and complex challenges to the bacterial host. There are few reports of co-production of molecular chaperones with membrane proteins in *E. coli *due to the paucity of reports of recombinant membrane protein produced successfully in the host in general. Amongst these, the expression and solubility of the HrcA repressor from *Helicobacter pylori *were dramatically increased upon induction of heat shock proteins by elevated temperature [109] while overexpression of GroESL led to significantly improved expression of the human liver cytochrome P450 2B6 [110] and a DnaK-DnaJ combination reduced inclusion body formation by the CorA bacterial magnesium transporter [66]. While the present body of literature does not make a particularly compelling case for adding chaperones to membrane protein production experiments in *E. coli*, screening of their influence in such set-ups is clearly advisable due to the simplicity and low cost of the approach, and their potential benefits on the passage through the cytoplasm and/or periplasm of these often highly hydrophobic and difficult-to-express proteins.

### Small heat shock proteins

Small heat shock proteins (sHsps) are a ubiquitous group of proteins that tend to exist *in vivo *as macromolecular complexes, the stoichiometry of which varies between different sHsps (reviewed in [111]). They bind non-native proteins with a high degree of promiscuity in an ATP-independent manner and their slowness of substrate release has led to speculation that they may function primarily as reservoirs of unfolded protein in times of stress. It is also likely that, upon removal of the physiological stress, they interact with other chaperones such as the Hsp70 group, leading to peptide release and ATP-dependent folding [111,112]. Their native activity has led to some interest recently in their potential usefulness in increasing the solubility of heterologous proteins in *E. coli*.

*E. coli *IbpA and IbpB have been demonstrated to protect misfolded proteins from irreversible aggregation and are thought to help to resolubilise protein aggregates [113-115]. Overproduction of IbpAB led to increased production of *E. coli *malic enzyme, enhanced green fluorescent protein, and human IGF-I_f_, interferon γ and interleukin-12 β chain by 1.3- to 2-fold in the *E. coli *cytoplasm [116], as well as increased soluble yields of 17 of 23 proteins, many difficult to express, in an extensive investigation of overproduction of the entire network of major *E. coli *cytosolic chaperones ([47] see also [46]). Increased yields of soluble proteins were also obtained upon co-overproduction of IbpAB with DnaK-DnaJ-GrpE, ClpB and GroESL, albeit only to levels attainable upon addition of the heat shock-inducer benzyl alcohol [117]. Conversely, overproduction of IbpAB could not suppress inclusion body formation by preS2-S'-β-galactosidase [118], while successful production of a human GTPase activating protein, which led to cell lysis under standard expression conditions, could be achieved *only *in a Δ*ibpAB E. coli *strain [119]. Co-production of hexadecameric murine Hsp25, meanwhile, fused to an *ompA *signal peptide, increased the amount of functional tPA variant in the *E. coli *periplasm by 125-fold [51] but there was no increase in the periplasmic yield of native proinsulin in the same study.

de Marco and co-workers recently presented an extensive evaluation of the effects of coproduction of IbpAB in association with Hsp70, Hsp60 and ClpB proteins [46,47]. In their approach, protein production (and chaperone co-production) was followed by a period of inhibition of protein synthesis to allow chaperone-mediated refolding of misfolded or aggregated polypeptides. The overall effect of co-overproduction of IbpAB was an increase in the solubility of 20 of 23 proteins tested, including 12 that could not be produced in soluble form in the absence of IbpAB [47].

### Miscellaneous accessory molecules

In additional to "conventional" chaperones, the ability of a variety of accessory proteins to improve recombinant protein production and/or activity in *E. coli *has been evaluated. One of the most common of these is thioredoxin (Trx), as discussed later in the context of disulfide bond metabolism. ClpB, meanwhile, is a large, star-shaped hexameric molecule that interacts with the DnaK chaperone system in a currently unresolved manner to disaggregate insoluble polypeptide aggregates (reviewed in [120]). It is postulated to unfold and pass polypeptides through its central, 13-Å channel [121,122] and is an obvious candidate for overproduction given its recognised ability to disaggregate polypeptide aggregates. This potential is borne out by the observation that, while various combinations of Hsp60 and Hsp70 proteins could *dissolve *macromolecular aggregates of human basic fibroblast growth factor, this typically was not concomitant with increased solubility of the target unless ClpB was also overproduced [44]. Overproduction of tRNA molecules specific for *E. coli *rare codons, often carried out in combination with conventional chaperone co-production, has also been commonly used to increase yields of proteins from species with a codon bias significantly different from that of *E. coli*, including archaeabacteria [77], *Plasmodium *[123], viruses [124] and eukaryotes [125].

A further approach to chaperoning heterologous proteins in *E. coli *is to provide their native chaperone or accessory protein where possible, as in the case of co-production of the rubisco-specific chaperone RbcX with *Synechococcus *ribulose-1,5-biphosphate carboxylase/oxygenase [126]. The activity of 17α-hydroxylase-C_17,20_-lyase (P450cl7) also increased 100-fold upon co-production of rat NADPH-cytochrome-P450-reductase [127] while the solubility of human retinoic acid receptor [128] and α-subunits of human haemoglobin [129] increased upon co-production of their binding partner and specific stabilising chaperone, respectively.

## Secretion from the cytoplasm

Proteins destined for the non-reducing environment of the periplasm are most commonly secreted using the Sec (*sec*retion) family [130]. Cytosolic SecB associates with unfolded proteins in an ATP-independent manner and delivers them to SecA, the site of preprotein entry into the membrane-bound translocase [131,132]. Translocation is achieved through the SecEY complex, which forms a pore through which the preprotein passes [133,134], and involves the action of SecG, which "lubricates" the pore for insertion of a SecA domain [133,135]) and SecD and SecF, which prevent reverse translocation of the preprotein [133].

**Figure 2:**
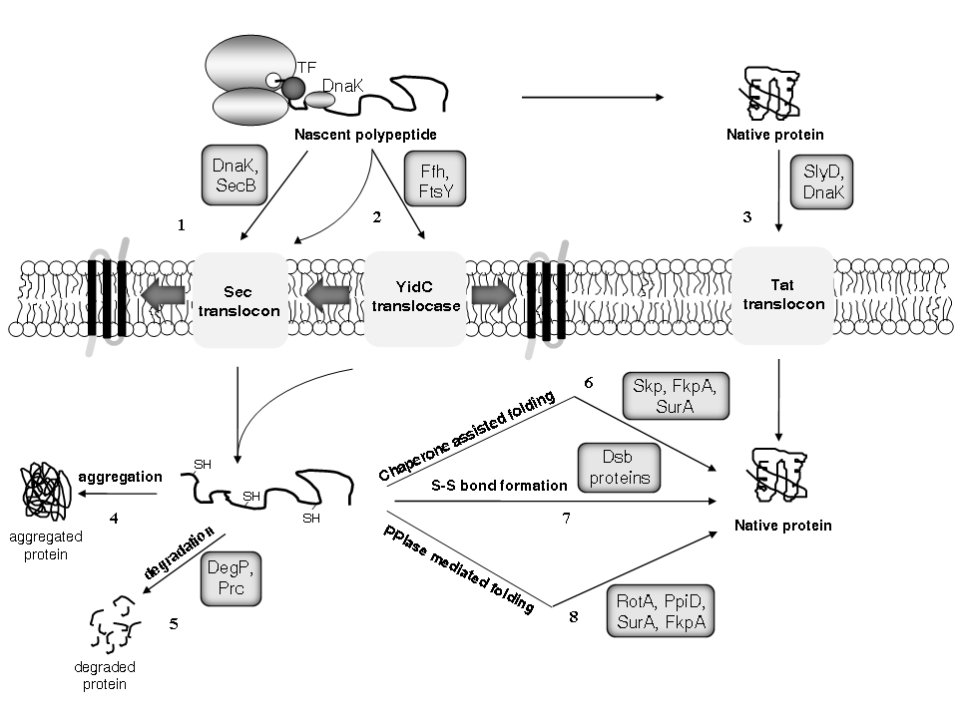
**Membrane translocation and periplasmic folding in *E. coli***. Most polypeptides cross the cytoplasmic membrane in an unfolded conformation using the Sec translocase (1), following delivery to SecA at the inner surface of the membrane by DnaK or SecB chaperones. Polypeptides with highly hydrophobic signal sequences or transmembrane domains may, however, be recognised by Ffh which, together with its FtsY receptor, can target the polypeptide to either the Sec machinery or to the YidC translocase (2). Alternatively, the twin-arginine translocation (Tat) machinery is responsible for the translocation of already folded proteins (3), typically with bound metal cofactors. After cleavage of the leader peptide upon crossing the membrane, partially folded proteins may (4) aggregate, (5) be degraded by periplasmic proteases, or fold into their native state, often with the assistance of periplasmic chaperones (6) and/or folding catalysts such as disulfide bond metabolising enzymes (7) or peptidyl-prolyl *cis*-*trans *isomerases (8).

In addition to the *sec *pathway, a less well characterised twin-arginine translocation (*tat*) pathway of membrane translocation also exists [136]. The essential components of this pathway are the TatA, TatB and TatC integral membrane proteins, which recognise a critical twin arginine motif in the N-terminal signal sequence of polypeptide substrates. Unlike the *sec *system, the Tat pathway can transport proteins across the cytoplasmic membrane in a fully folded state (Figure 2; [136,137]). Furthermore, two distinct systems, the first employing a homologue of the eukaryotic signal recognition particle called the *f*ifty-*f*our *h*omologue (Ffh; [138]) and its FtsY receptor and the second the 61-kDa cytoplasmic protein YidC [139], are involved primarily in targeting integral membrane (pre)proteins to the inner membrane in *E. coli*. The possible membrane translocation routes of recombinant polypeptides, and their subsequent folding in the periplasm, are represented in Figure 2.

### Improving the process of secretion

While manipulation of the Sec pathway initially concentrated largely on the SecEY tranlocase, the disappointing results led to most studies focussing instead on the SecA and SecB proteins that deliver polypeptides to the translocase. Even then, results remained unspectacular: SecB overproduction resulted in increased solubility and a higher yield of a penicillin acylase, though enzyme activity was not increased [140], while SecB and SecF overproduction led to 3- and 2-fold increases, respectively, in the periplasmic activity of a penicillin amidase [141].

Comparatively little analysis of *tat *gene overexpression has been carried out, though overexpression of *tatABC*, in combination with manipulation of physiological conditions, led to a 20-fold increase in the level of a green fluorescent protein that otherwise rapidly saturates the *tat *translocation machinery [142]. Co-expression of phage shock protein A (PspA) can also relieve saturation of protein export via this pathway [143] while Han and co-workers [116] demonstrated that knocking out the sHsps IbpA and IbpB led to enhanced secretion of enhanced green fluorescent protein (EGFP) from *Aequorea victoria *via both the *sec *and *tat *secretion pathways. The recent demonstration that DnaK and SlyD chaperones serve as general Tat signal-binding proteins [144,145], in tandem with the promising outcomes of the limited investigation of the pathway to date, is likely to focus increased attention on using the *tat *machinery to improve periplasmic expression over the coming years.

Meanwhile, in the only evaluation of Ffh overproduction reported to date, co-overexpression of *ffh*/*ffs *(the latter encoding 4.5S RNA) or *ffh*/*ffs*/*ftsY *with the bacterial inner membrane magnesium transporter CorA led to reduced expression of CorA and failed to prevent inclusion body formation [66].

Overall, while *E. coli *strains that allow formation of disulfide bridges in the cytoplasm are now available, thus negating the need for secretion of disulfide-containing recombinant proteins, there is little evidence that the secretion process limits the production of most heterologous proteins. Instead, the bottleneck for production is usually more likely to involve maintenance of polypeptides in a non-aggregated, translocation-competent form in the cytoplasm or in avoidance of aggregation in the periplasm subsequent to membrane translocation.

## Folding in the periplasm

Following membrane translocation, folding of the heterologous polypeptide takes place in the periplasmic space (Figure 2). While disulfide bond formation and peptidyl-prolyl *cis*-*trans *isomerisation can occur here, no general molecular chaperones that prevent non-productive folding reactions had been identified until relatively recently, when a variety of molecules such as Skp, FkpA, SurA and DegP were independently isolated and characterised.

Skp is an *E. coli *periplasmic *s*eventeen *k*iloDalton *p*rotein that has been found to assist the production of difficult-to-express antibody fragments in particular. Skp co-production led to delayed cell lysis and improved production of single-chain antibody fragments (scAbs; [146]), higher yields and increased antigen binding activity of scFvs [147], improved functional production of phage-displayed scFvs [148] and improved production and secretion of a Fab fragment [149]. Meanwhile, a signal sequence-less Skp has also been used to increase the yield of active Fab fragment in the cytoplasm of an *E. coli *Origami strain [19]. Skp co-production has also been utilised, in combination with protein engineering, to achieve high-level secretion of three single-chain T cell receptors [150], which, though structurally similar to antibody fragments, have traditionally proven difficult to produce in active form in *E. coli*. Skp has been also found to enhance the *E. coli *cell surface display of a yellow fluorescence protein by reducing the extracytoplasmic stress and, thus, improving cell physiology [151].

DegP is a periplasmic protease that is proposed to undergo a shift to function as a chaperone at low temperatures, though it has recently been demonstrated to retain proteolytic activity as low as 20°C [152]. It is active in the same pathway as Skp, with SurA active in a parallel chaperone pathway. DegP overproduction has been found to reduce inclusion body formation in the periplasm and to increase the activity of penicillin acylase in *E. coli *[153,154], while SurA and FkpA chaperones possess PPIase activity in addition to their chaperone functions and are discussed later.

### Manipulation of the disulfide bond metabolic machinery

Formation of stable disulfide bonds is confined to the oxidising periplasmic environment in *E. coli*, where disulfide bond formation, reduction and isomerisation are catalysed by the Dsb (*d*i*s*ulfide *b*ond formation) family in a thiol-disulfide exchange between their active site Cys-X-X-Cys cysteines and those of the target protein (reviewed in [155]). DsbA catalyses disulfide bond formation by transferring its own active site disulfide to the target protein, leaving DsbA in a reduced form, whereupon it is reoxidised by the cytoplasmic membrane-bound DsbB. DsbB in turn passes its electrons to the respiratory chain to regenerate its own oxidised state. "Shuffling", or isomerisation, of incorrectly-formed disulfide pairs is carried out by DsbC, which is maintained in its reduced form by the cytoplasmic membrane-bound DsbD [156,157]. Other Dsb proteins in *E. coli *include DsbE, which is required for cytochrome c biogenesis, and DsbG, which catalyses disulfide bond formation but is an inefficient catalyst of disulfide rearrangement.

DsbA and DsbC in particular are commonly co-produced with disulfide-linked recombinant proteins in the periplasm, an approach which has met with considerable success. DsbA overproduction has led to increased functional yields of numerous proteins, such as heat-labile enterotoxins [158], as well as increased solubility of human leptin [159] and cyclohexanone monooxygenase [35]. DsbC has now become the Dsb protein of choice, however, particularly when producing proteins with complex disulfide patterns such as tissue plasminogen activator [160,161] or *Ragi *bifunctional inhibitor [162], due to its potential to rescue misfolded or partially aggregated polypeptide through its isomerisation activity. In one of the few direct comparisons of the effects of the two enzymes, the expression level of insulin-like growth factor-I increased 2-fold and 1.7-fold, respectively, with DsbA and DsbC [163]. Zhang and co-workers [164] also reported improved solubility of a diverse range of eukaryotic proteins upon fusion with a DsbA variant lacking active site cysteine residues, however, which raises the question of whether Dsb proteins aid expression of recombinant proteins due to their catalytic properties or – as often in the case of PPIases (see below) – due to a chaperoning capability.

Co-production of DsbBD, DsbAB and DsbAC pairs also all led to higher functional yields of glutamate racemase (GluR; [86]) – despite the fact that GluR, while possessing 5 cysteine thiol groups, contains no disulfide bridges in its native structure. Conversely, expression of human nerve growth factor [165], horseradish peroxidase [105,166] and brain-derived neurophilic factor [167] are all more efficient in the presence of DsbABCD than individual or pairs of family members.

A number of examples also exist in the literature of co-production of thioredoxin (Trx), a small (12 kDa) protein with a Cys-X-X-Cys active site motif, which too is typically co-produced with cysteine-rich polypeptides. This approach allowed functional production of a snake venom thrombin-like enzyme, which in the absence of Trx was not detectable in the cytoplasm [168], while Trx also dramatically increased the solubility of mouse c-Myb, cAMP response element-binding protein 1, p53 tumour suppressor gene product, adenovirus oncogene product E1A, *Xenopus mos *proto-oncogene product and the tyrosine kinase Lck, all of which were otherwise produced in inclusion bodies [65]. Thioredoxin co-production has also been demonstrated to increase by 3–4 fold the yield of functional ribotoxin α-sarcin in the *E. coli *periplasm [169]. In addition to its use as a separate, co-produced protein, thioredoxin is commonly employed as a fusion tag to improve the expression or solubility of heterologous proteins.

While stable disulfide bridges cannot normally be formed in the strongly reducing *E. coli *cytoplasm, there are increasing attempts to produce disulfide-containing heterologous proteins there, usually by blocking either or both of the thioredoxin/thioredoxin reductase and glutathione/glutaredoxin reducing pathways. Co-expression of a leaderless *dsbC *in such an *E. coli *background has greatly enhanced production of antibody fragments [19] and of proteins with complex disulfide patterns, such as tPA [161], thrombin-like enzyme calobin [168] and snake venom fibrolase [170], such that screening of production of cysteine-rich recombinant proteins that are particularly prone to aggregation is well-advised in *E. coli trx *or *gor *mutant strains.

### Overproduction of periplasmic PPIases

PPIases exist in three distinct families in *E. coli*: cyclophilins, whose isomerase (rotamase) activity is reversibly inhibited by cyclosporin A and which are thought to be essential in processes such as protein folding and subcellular trafficking; FKBPs (*FK*506 *b*inding *p*roteins), which are inhibited by FK506 and rapamycin and include the periplasmic FkpA and cytoplasmic metal-binding SlyD and trigger factor (discussed above), which is novel amongst FKBPs in exhibiting no affinity for FK506; and parvulins, including the periplasmic SurA and membrane-bound PpiD, which are insensitive to immunosuppressors but irreversibly inhibited by juglone and have roles in the folding of outer membrane proteins [10]. The three families exhibit limited sequence and structural similarity but share a high catalytic activity and a relatively low affinity for nonstructured peptides [171].

Of the 4 PPIases identified in the *E. coli *periplasm – PpiA (RotA), PpiD, FkpA and SurA – mutations in PpiA have been shown to have no effect on the folding of periplasmic and outer membrane proteins [172] and PpiA overproduction has failed to improve expression of scFv fragments or a single-chain TcR [173,174]. There have been no reports of overproduction of PpiD as an approach to improving heterologous protein production in *E. coli *but the recent report that it interacts with proteins exiting the SecYEG translocon [175] suggests it may be a promising target for overexpression with problematic secretory proteins. SurA, also a parvulin, was found to improve the folding of unstable or aggregation-prone proteins in the periplasm [176] but failed to help production of a scFv fragment [173]. Meanwhile FkpA, which like trigger factor possesses both chaperone and PPIase activity, enhanced production of a wide range of scFv fragments by up to 10-fold when overproduced [173], while its fusion to various scAb fragments also led to increased solubility and higher functional yields [177]. FkpA co-production also led to increased hydrolysis of ampicillin by a catalytic scFv [178] and enhanced the production of penicillin acylase [179].

## Fusion tags

A related approach to improving recombinant protein solubility, though outside the scope of this review, is the use of fusion tags, *e.g*. thioredoxin, maltose-binding protein, glutathione *S*-transferase and, more recently, *E. coli *stress-responsive proteins such as RpoS [180], SlyD [181], and PotD and Crr [182]. This strategy generally results in reliably high protein yields and can simplify purification due to the affinity of certain fusion partners for a particular ligand. It also leads to considerations about how the fusion partner may affect folding or activity and the requirement for its precise removal, but the general approach has met with considerable success in increasing protein solubility, as reviewed elsewhere [183,184].

## Overview

**Figure 3:**
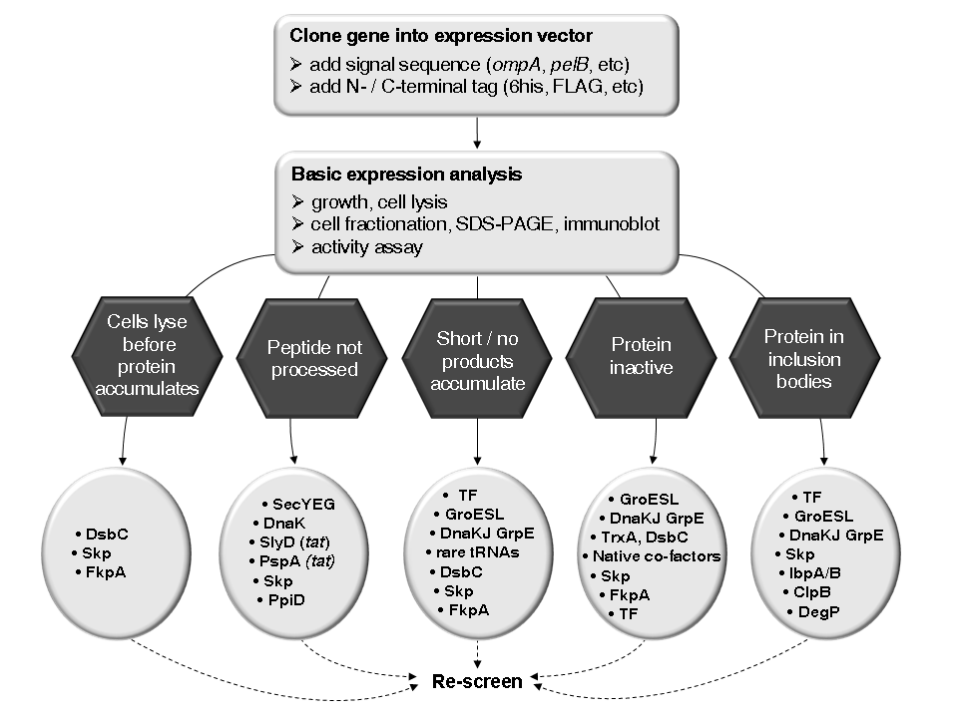
**Strategy for selection of molecular chaperones and folding catalysts for co-production analyses**. Following production of a recombinant protein in *E. coli*, analysis of cell growth, protein solubility and subcellular location, macromolecular state and activity provide some insight into the limiting step in the folding and production process. This Figure shows the major bottlenecks typically encountered (in hexagons) during production of a difficult-to-express recombinant target and identifies the co-production strategies that have been most successful in overcoming these bottlenecks to date (corresponding ovals).

Though there are currently no rigid criteria by which one might identify *in silico *the "correct" chaperone(s) to overcome a particular bottleneck in protein production, the present review identifies the co-production strategies that have most successfully overcome the major problems limiting recombinant protein yields in *E. coli *to date. Based on the current state-of-the-art, therefore, we summarise in Figure 3 some targeted interventions that might be used to focus chaperone screening strategies on already proven approaches, thus increasing the chances of successful production of well-behaved, active protein.

## Concluding remarks

Co-production of molecular chaperones and folding catalyst improves – very greatly in many cases – the production of many heterologous proteins in *E. coli*. While there is still no panacea for folding problems, nor even a rational method to identify the optimal candidate(s) for co-production with a target of interest, researchers are increasingly turning to chaperone co-production systems, available both commercially and non-commercially, as their first port of call when looking to overcome folding bottlenecks. While such a multi-chaperone screening approach can bear fruit quickly and relatively painlessly, detailed mechanistic studies of individual folding modulators remain essential in order to better understand their molecular mechanisms for greater, longer term practical benefits to the field of *E. coli *recombinant protein production.

Finally, though the merit of co-expression of some eukaryotic chaperones in *E. coli *is long recognised [185,186], the immense potential of chaperones from extremophilic species has only recently begun to be mined. Successes to date with GroESL homologues from *Oleispira antarctica *[187] and a novel trigger factor from another psychrophile, *Psychrobacter frigidicola *[188], suggest that these studies may represent the beginning of a new era in chaperone-assisted production of recombinant proteins in *E. coli*.

## Competing interests

The authors declare that they have no competing interests.

## Authors' contributions

All authors contributed equally to this manuscript, and read and approved the final version.
